# In the March 2015 issue

**DOI:** 10.1590/S1980-57642015DN91000001

**Published:** 2015

**Authors:** Ricardo Nitrini

**Affiliations:** Editor-in-Chief

In this issue we are publishing three reviews, eight original papers, one history note and one case report.

**Diehl-Rodrigues and Grinberg** reviewed the literature on argyrophilic grain disease, a highly frequent yet largely unknown tauopathy. The authors provide an indepth overview of the neurologic and neuropsychiatric symptoms and of the neuropathological features of this condition.

**Bahia and Pereira** reviewed the relationship between obstructive sleep apnea and neurodegenerative diseases, highlighting that neurodegenerative diseases can lead to apnea and that apnea may accelerate the degenerative process in the nervous system. The relevance of the treatment of obstructive sleep apnea is emphasized.

**Bragança et al.** reviewed the neural basis of synesthesia and the possible role in non-synesthetes, taking into account musical meaning, where memories, images and emotions are activated by the perception of music.

**Machado and Reppold** conducted a systematic review of the literature to investigate the effects of deep brain stimulation on the motor and cognitive symptoms of patients with Parkinson disease. There are clear positive effects on motor symptoms but establishing conclusions for cognitive functions was hampered by the absence of a gold-standard protocol for neuropsychological assessment.

**Custodio et al.** Investigated the costs of dementia in Lima, Peru, in a retrospective study including 136 out-patients. The cost of dementia was found to be very high for patients' families.

**Ferretti et al.** reported preliminary data on costs of dementia in a Brazilian setting. In a cross-sectional study, where 93 caregivers were interviewed, indirect costs were higher than other previous Latin American studies.

**Brigola et al.** performed a systematic review of the literature, exploring the association of subjective memory complaints with cognitive impairment, depression and other neuropsychiatric disorders. Depression and cognitive impairment were associated with subjective memory complaints and their occurrence seems to increase the chance of dementia.

**Oliveira et al.** investigated the association between predicted performance on a memory test, an aspect of metamemory, and objective performance. No difference was found between men and women or between groups with different educational levels.

**Camargo et al.** compared several characteristics of patients seen in public and private outpatient reference services in Southern Brazil. Patients seeking the public health service reached the system at more advanced stages of disease and had less access to medical care.

**Ward et al.** investigated the presence of apraxia in patients with Alzheimer's disease, mild cognitive impairment and controls using eight subitems of the Cambridge Cognitive Examination (CAMCOG). Differences were found between the three groups, showing that testing for the presence of apraxia may be useful for the diagnosis of these conditions.

**Lampert and Rosso** evaluated the prevalence of depressive symptoms in a long-stay nursing home for elderly women in Southern Brazil. Scores higher than five on the Geriatric Depression Scale - indicative of significant depressive symptoms - were found in 32.3% of the 142 elderly women. The psychosocial factors involved in this high prevalence of depression warrant further studies.

**Engelhardt and Grinberg** reviewed, in a history note, the relevant contributions of Alois Alzheimer to our current understanding of vascular cognitive impairment and vascular dementia.

**Machado-Porto et al.** reported a case of hydrocephalic dementia due to neurocysticercosis that also presented racemose cysts. Improvement was associated with ventricular derivation and chronic use of antihistamines.

**Ricardo Nitrini** Editor-in-Chie

